# Congenital Hernia and Hydrocele

**Published:** 1888-09

**Authors:** 


### THE CURE OF CONGENITAL HERNIA AND HYDROCELE.

At the close of a lecture on this subject Professor L. G. Richelot draws the following conclusions:

1. In congenital inguinal hernias, as in all others, resection of the sac is one of the conditions of the radical cure.

2. Total resection of the inguinal sac is always possible, whether in kelotomy for strangulation or in radical cure made deliberately.

3. Resection of the vagino-peritoneal canal is always possible in congenital hernias without ectopia, as it is fixed at the external inguinal ring or sufficiently movable to descend. It may be made so as to preserve the testicle and close the vaginalis around it. It is a delicate operation, but has no great difficulties.

4. Resection of the vagino-peritoneal conduit, without hernia, is also easily done, and under the same conditions, when a reducible hydrocele enables the operator to recognize the congenital disposition. It closes the serous canal that may become a sac, and has the value of a preventive radical operation.

6. A benign operation, and without mutilation, that delivers a young man from a great infirmity, is one that should be recommended.—*L'Union Médicale*, No. 52, 1888.

